# *Blechnum Orientale *Linn - a fern with potential as antioxidant, anticancer and antibacterial agent

**DOI:** 10.1186/1472-6882-10-15

**Published:** 2010-04-30

**Authors:** How Y Lai, Yau Y Lim, Kah H Kim

**Affiliations:** 1 School of Science, Monash University Sunway Campus, Bandar Sunway, 46150 Petaling Jaya, Selangor, Malaysia; 2 Department of Physiology, Faculty of Medicine, University of Malaya, 50603 Kuala Lumpur, Malaysia

## Abstract

**Background:**

*Blechnum orientale *Linn. (*Blechnaceae*) is used ethnomedicinally for the treatment of various skin diseases, stomach pain, urinary bladder complaints and sterilization of women. The aim of the study was to evaluate antioxidant, anticancer and antibacterial activity of five solvent fractions obtained from the methanol extract of the leaves of *Blechnum orientale *Linn.

**Methods:**

Five solvent fractions were obtained from the methanol extract of *B. orientale* through successive partitioning with petroleum ether, chloroform, ethyl acetate, butanol and water. Total phenolic content was assessed using Folin-Ciocalteu's method. The antioxidant activity was determined by measuring the scavenging activity of DPPH radicals. Cytotoxic activity was tested against four cancer cell lines and a non-malignant cell using MTT assay. Antibacterial activity was assessed using the disc diffusion and broth microdilution assays. Standard phytochemical screening tests for saponins, tannins, terpenoids, flavonoids and alkaloids were also conducted.

**Results:**

The ethyl acetate, butanol and water fractions possessed strong radical scavenging activity (IC_50 _8.6-13.0 μg/ml) and cytotoxic activity towards human colon cancer cell HT-29 (IC_50 _27.5-42.8 μg/ml). The three extracts were also effective against all Gram-positive bacteria tested: *Bacillus cereus, Micrococcus luteus*, methicillin-susceptible *Staphylococcus aureus *(MSSA), methicillin-resistant *Staphylococcus aureus *(MRSA) and *Stapylococcus epidermidis*(minimum inhibitory concentration MIC 15.6-250 μg/ml; minimum bactericidal concentration MBC 15.6-250 μg/ml). Phytochemical analysis revealed the presence of flavonoids, terpenoids and tannins. Ethyl acetate and butanol fractions showed highest total phenolic content (675-804 mg gallic acid equivalent/g).

**Conclusions:**

The results indicate that this fern is a potential candidate to be used as an antioxidant agent, for colon cancer therapy and for treatment of MRSA infections and other MSSA/Gram-positive bacterial infectious diseases.

## Background

The World Health Organization estimates that 80% of the world's inhabitants rely mainly on traditional medicines for their health care [1]. Many medicinal plants have proved to successfully aid in various ailments leading to mass screening for their therapeutic components. Today, the search for natural compounds rich in antioxidant, anticancer and antimicrobial properties is escalating due to their medicinal importance in controlling many related chronic disorders such as cancer and cardiovascular diseases. Antioxidants aid in the prevention by scavenging the excess free radicals in the body. Cancer is currently a leading cause of death and growing evidence relates its occurrence to the oxidative damage to DNA, proteins and lipid in the body [2]. It has been estimated that approximately two-thirds of anticancer drugs approved worldwide up to 1994 were derived from plant sources [3]. The rapid emergence of multiple drug resistant strains of pathogens to current antimicrobial agents has generated an urgent intensive search for new antibiotics from medicinal plants. Many medicinal plants have been screened extensively for their antimicrobial potential worldwide [4-6]. *Blechnum orientale *Linn. (*Blechnaceae*) is known as the Centipede fern or 'paku ikan' (by the Malays) or 'Kuan Chung' (by the Chinese). It is natively distributed in Malaysia, in many Asia countries, Australia and Pacific islands. This fern can reach a height of 2.5 m and commonly grows on exposed low hillsides to 1500 m altitude mountains. Its leaf is traditionally used as poultice to treat boils, blisters or abscesses and sores, as a diaphoretic, for stomach pain, urinary bladder complaints and sterilization of women [7-9]. The leaf is also boiled and eaten as vegetable by the natives [10,11].

As part of an ongoing search for natural antibacterial and antioxidant agents from medicinal ferns, we found that the methanol leaf extract of *B. orientale *possessed the strongest potential as broad spectrum bactericidal [12]. Subsequently, we fractionated the methanol extract with increasing polarity of solvents in order to facilitate isolation of bioactive compounds. In this study, we report our findings on the antioxidant, antibacterial and cytotoxic activities of five solvent fractions obtained from the methanol extract of this fern. To the best of our knowledge, this is the first published report on its cytotoxic activity towards human colon cancer cell HT-29 and bactericidal activity towards methicillin-resistant *Staphylococcus aureus *MRSA.

## Methods

### Plant material and extraction

*Blechnum orientale *Linn. was obtained from Putrajaya Botanical Garden, Kuala Lumpur. The identity was confirmed by plant taxonomist Anthonysamy S., formerly from University Putra Malaysia and currently a consultant with the landscape consulting firm, Aroma Tropic Limited, Kuala Lumpur. A voucher specimen (LAA007) was deposited at the Herbarium of Monash University Sunway Campus.

Three batches of leaves were collected in April, August and November of 2008. Each batch of the freeze-dried powdered leaves of *B. orientale *(totalling 1.7 kg from three batches) were repeatedly extracted with methanol (1:10, w/v) until the extracts were light coloured. The extract was evaporated under reduced pressure to dryness as a dark green mass (total 292 g) with 17% yield. Each batch of the mass was suspended in distilled water (1:10, w/v). The suspension was successively partitioned with petroleum ether 40-60°C, chloroform, ethyl acetate and *n*-butanol. The solvents were evaporated to dryness under reduced pressure. The extracts obtained were eventually freeze-dried to remove any residual water, yielding petroleum ether fraction (PEf), chloroform fraction (Cf,), ethyl acetate fraction (EAf), butanol fraction (Butf) and water fraction (Watf). The yield of each fraction (Table 1) was expressed as a weight percentage of the dry leaves. All the samples were stored at -70°C until used.

### Total phenolic content (TPC)

Total phenolic content (TPC) in extracts was determined using the procedure previously described [12]. Extract solution (0.3 ml, in triplicate) was mixed with 1.5 ml of 10% Folin-Ciocalteau's reagent and 1.2 ml of 7.5% (w/v) sodium carbonate. The mixture was kept in the dark for 30 min and absorbance was measured at 765 nm. The gallic acid standard curve used was y = 0.01078× (R^2 ^= 0.9996) where y is absorbance at 765 nm and × is the concentration of gallic acid in mg/l. TPC was expressed as mg gallic acid equivalent (GAE)/g extract.

### DPPH radical scavenging activity

The DPPH assay was performed according to the method described by Wang *et al*. [13]. Ascorbic acid, α-tocopherol, BHT and Trolox-C were used as positive controls. Various dilutions of the extract in methanol (100 μl of 2-1000 μg/ml, in triplicate) were added to 100 μl of DPPH (200 μM) in a 96-well plate. The mixture was left in the dark for 30 min, before reading the absorbance at 517 nm. The control consisted of methanol instead of the sample. The percentage radical scavenging activity was calculated as follows: **s**cavenging (%) = (A _control_- A_sample_)/A_control _× 100. The result was expressed as IC_50_, the concentration of the extract to scavenge 50% of the DPPH radical.

### Cytotoxic activity

The human colonic adenocarcinoma HT-29, human colonic carcinoma HCT-116, human breast adenocarcinoma MCF-7, human leukemia K562 and liver Chang cells were obtained from Monoclonal Lab, Faculty of Medicine, University of Malaya. The cells were cultured in a humidified atmosphere at 37°C in 5% CO_2_. DMEM supplemented with 10% fetal calf serum, 1% (v/v) penicillin/streptomycin and 2 mM L-glutamine was used as the culture medium of MCF-7, K562 and liver Chang cells. RPMI-1640 supplemented with 10% fetal calf serum, 2 mM L-glutamine and 1% (v/v) penicillin/streptomycin was used for cell cultures of HT-29 and HCT-116.

Cytotoxicity was measured using the MTT (Thiazolyl Blue Tetrazolium Bromide) assay adapted from Tan *et al*. [14]. Briefly, 96-well plates were seeded with 180 μl of medium containing an appropriate number of cells (2000 for HCT-116, 3000 for MCF-7, 4000 for Chang liver, 5000 for K562 and 6000 for HT-29). The cells were allowed to adhere for 24 h. Extracts (20 mg/ml) were dissolved in 20% DMSO and diluted to 1 mg/ml with medium. Aliquots of 20 μL of the extracts at various concentrations (10 - 100 μg/ml) were added to each well. For the control, 20 μL of 1% DMSO in the medium was used instead of extract. The final concentration of DMSO was less than 1% and as the extracts were used in the dried form, there was no residual butanol or ethyl acetate in the Butf and EAf, respectively. Curcumin (Sigma) was used as positive control. After incubation for 72 h, 50 μl of 2 mg/ml MTT (Sigma) was added to each well. The plate was incubated for another 3 h. The medium of each well was pipetted out and 150 μl of DMSO was then added. Absorbance was read at 554 nm using a Bio-TEK Microplate Scanning Spectrophotometer. The cytotoxic effect was expressed as IC_50_, the concentration of extract that reduces cell viability to 50% of the control.

### Antibacterial assays

#### Bacterial strains and media

Ten strains of bacteria were obtained from stock cultures preserved at -70°C at the Microbiology Lab, School of Science, Monash University Sunway Campus. Five Gram-positive bacteria tested were *Bacillus cereus*ATCC14579, *Micrococcus luteus*ATCC4698, methicillin-susceptible *Staphylococcus aureus *MSSA ATCC25923, methicillin-resistant *Staphylococcus aureus *MRSA ATCC33591 and *Stapylococcus epidermidis *ATCC12228 and five Gram-negative bacteria tested were *Escherichia coli*ATCC25922, *Pseudomonas aeruginosa*ATCC10145, *Klebsiella pneumoniae*ATCC10031, *Salmonella choleraesuis *and *Enterobacter aerogenes*. All bacteria were grown on nutrient agar (Oxoid).

#### Disc diffusion test

Diameter of zone of inhibition was determined using the disc diffusion method as previously described [12]. A swab of the bacteria suspension containing 1 × 10^8 ^CFU/ml was spread onto petri plates containing Mueller-Hinton agar (MHA). Extracts were dissolved in methanol to final concentration of 10 mg/ml. Sterile filter paper discs (6 mm in diameter) impregnated with 1 mg of plant extracts were placed on the cultured plates. The plates were incubated at 37°C for 24 h. Methanol served as negative control while standard streptomycin (10 μg), oxacillin (1 μg) and vancomycin (30 μg) discs were used as positive controls. Antimicrobial activity was indicated by the presence of clear inhibition zones around the discs. The assay was repeated twice and mean of the three experiments was recorded.

#### Determination of minimum inhibitory concentration (MIC) and minimum bactericidal concentration (MBC)

Broth microdilution assay was used to determine the MIC and MBC [15]. The crude extract was dissolved in 50% DMSO at a concentration of 50 mg/ml which was then diluted with nutrient broth to 1 mg/ml. The ethyl acetate, butanol and water fractions were prepared in nutrient broth. Standard antibiotic vancomycin (Sigma) was prepared at concentrations of 0.48-31.25 μg/ml. Test sample (75 μl) of various concentrations (2-1000 μg/ml) was added into sterile 96-well plates. Bacterial cell suspension (75 μl) corresponding to 1 × 10^6^CFU/ml was added in all wells except those in columns 11. Wells of column 11 consisted of 1% DMSO and broth served as controls to check sterility while those in column 12 were filled with nutrient broth and bacterial suspension to check for adequacy of the broth to support bacteria growth. The plates were covered with sterile sealer and incubated at 37°C for 18-24 h. To indicate bacterial growth, 40 μl of 0.2 mg/ml p-iodonitroterazolium chloride (INT, Sigma) was added to each well and incubated for another 30 min [16]. Inhibition of bacterial growth was visible as a clear colourless well and the presence of growth was detected by the presence of pink-red color. The lowest concentration showing no colour change was considered as the MIC.

For determination of MBC, a loop of liquid from each well that showed no change in colour was streaked onto MHA and incubated at 37°C for 24 h. The lowest concentration that showed no growth was taken as the MBC. Experiments were done in triplicate and repeated twice.

### Phytochemical screening

Phytochemical tests for saponins, tannins, terpenoids, flavonoids and alkaloids were performed as previously described [4,17]. Dragendorff reagent was used for alkaloids, foam test for saponins, Mg-HCl and Zn-HCl for flavonoids, Salkowski test for terpenoids, and ferric chloride and gelatin for tannins.

### Statistical analysis

All data were expressed as mean ± SD. Statistical analyses were evaluated by one-way ANOVA followed by Tukey HSD test. Values with P < 0.05 were considered statistically significant.

## Results

### Total phenolic content (TPC) and antioxidant activity

Total phenolic content (TPC), based on three batches of fern collection, was determined using the Folin-Ciocalteau reagent and expressed in terms of mg gallic acid equivalent (GAE)/g extract. The order in decreasing TPC was EAf ≈ Butf (675-804) > > Crude (346) > Watf (260) > Cf (214) > PEf (154) (Table 1).

The antioxidant activity of the methanol crude extract and its various fractions, as measured by the ability to scavenge DPPH free radicals, was compared with those of four standards, namely ascorbic acid, trolox-C, α-tocopherol and butylated hydroxytoluene (BHT). The lower the IC_50_, the stronger is the scavenging activity. The scavenging activity in decreasing order was ascorbic acid (5.3 μg/ml) > EAf ≈ Trolox-C (8.7 μg/ml) > crude ≈ Butf ≈ Watf ≈ α-tocopherol (10.1 -13.0 μg/ml) > BHT (17.2 μg/ml) > PEf (26.5 μg/ml) > Cf (37.5 μg/ml). It is clear that EAf was most effective with activity equal to Trolox-C and greater than α-tocopherol and BHT, while Butf and Watf were comparable with α-tocopherol.

### Cytotoxic activity

Table 2 summarizes the cytotoxic activity of the extracts. Due to difficulty in the solubility, the assay was not conducted on PEf and Cf. Considerable cytotoxic activity against the colonic adenocarcinoma cell HT-29 was detected in three fractions: Butf (IC_50 _27.5 μg/ml) > Watf (IC_50 _33.4 μg/ml) > EAf (IC_50 _42.8 μg/ml), although weaker compared to that shown by curcumin (IC_50 _5.4 μg/ml). Butf displayed weak cytotoxicity towards human colonic carcinoma cell HCT-116 (IC_50 _72.7 μg/ml). The fractions were not cytotoxic towards the other cancer cells MCF-7 and K562 as well as the liver Chang cell (IC _50 _> 100 μg/ml).

**Table 1:** Percentage yield, DPPH radical scavenging activity and total phenolic content of *Blechnum orientale *extracts

Sample	*Yield(g/100 g dry leaves)	**DPPH IC_50_(μg/ml)	**Total phenolic content (mg GAE/g dry weight)
Crude	17.2 ± 1.6	10.9 ± 1.6^a^	346 ± 48
PEf	6.3 ± 0.6	26.5 ± 2.2^b^	154 ± 10
Cf	0.1 ± 0.0	37.5 ± 3.0^c^	214 ± 14
EAf	1.6 ± 0.7	8.6 ± 0.5^d^	804 ± 72
Butf	2.7 ± 0.2	10.1 ± 1.1^a^	675 ± 62
Watf	6.5 ± 0.4	13.0 ± 1.3^a^	260 ± 9
Ascorbic acid		5.3 ± 0.1^e^	
Trolox-C		8.7 ± 0.1^d^	
α-Tocopherol		12.0 ± 0.7^a^	
BHT		17.2 ± 0.2^f^	

*Percentage yield is expressed as mean ± SD from 3 batches of collection.

** Results are mean ± SD from 3 batches of fern collection and 3 sets of experiments conducted on each batch (n = 9).

Cf = chloroform fraction; EAf = ethyl acetate fraction; Butf = butanol fraction; Watf = water fraction. Ascorbic acid, trolox-C, α-tocopherol and BHT (butylated hydroxytoluene) were used as standards.

For each column, values followed by the same letters are not statistically different (P > 0.05).

**Table 2:** Cytotoxic activities of water, butanol and ethyl acetate fractions of *Blechnum orientale*

	Cytotoxic activity IC _50_(μg/ml)			
Cell lines	EAf	Butf	Watf	Curcumin
HT-29	42.8 ± 6.2	27.5 ± 1.4	33.4 ± 1.9	5.4 ± 0.1
HCT-116	>100	72.7 ± 4.1	93.9 ± 3.2	5.5 ± 0.1
MCF-7	>100	>100	>100	n.t.
K562	>100	>100	>100	n.t.
Chang	>100	>100	>100	n.t.

Results are expressed as mean ± SD from three sets of experiment, each set in triplicate.

EAf = ethyl acetate fraction, Butf = butanol fraction, Watf = water fraction.

*Curcumin was used as positive control; n.t. denotes not tested.

**Table 3:** Inhibition zone diameter of *Blechnum orientale *extracts and standard antibiotics against five Gram-positive bacteria^a^

	^b^Inhibition zone diameter (mm)				
	*B. cereus*	*M. luteus*	MSSA	MRSA	*S. epidermidis*
^c^Crude	9.5	11.3	9.5	10.5	9.0
^c^PEf	7.0	7.5	7.9	8.0	-
^c^Cf	7.5	7.8	-	8.5	-
^c^EAf	11.0	13.8	10.8	13.0	9.1
^c^Butf	11.0	14.3	12.0	13.0	11.3
^c^Watf	10.3	12.8	10.5	11.5	9.8
^d^Streptomycin	18.0	24.0	16.6	-	-
^d^Vancomycin	18.0	30.0	17.5	20.0	19.5
^d^Oxacillin	8.0	48.0	22.3	-	25.5

Results are mean from three sets of experiments, each set in triplicate.

^a ^No activity was found against Gram-negative bacteria (*Escherichia coli, Pseudomonas aeruginosa, Enterobacter aerogenes, Salmonella choleraesius; Klebsiella pneumoniae*). Data not shown.

^b ^Inhibition zone diameter includes diameter of disc (6 mm); (-): not active

^c ^Extracts were tested at 1 mg/disc

^d ^Reference antibiotics discs: Streptomycin 10 μg; vancomycin 30 μg; oxacillin 1 μg

**Table 4:** Minimum inhibitory concentration (MIC in μg/ml) and minimum bactericidal concentration (MBC in μg/ml) of *Blechnum orientale *extracts

	*B. cereus*	*M. luteus*	MSSA	MRSA	*S. epidermidis*
	**Minimum Inhibitory concentration (MIC, μg/ml)**				
Crude	125	62.5	125	125	125
EAf	250	15.6	250	250	250
Butf	62.5	15.6	62.5	62.5	125
Watf	62.5	31.3	62.5	62.5	62.5
Vancomycin	1.9	0.5	1.9	1.9	1.9
					

	**Minimum bactericidal concentration (MBC, μg/ml)**				
Crude	125	62.5	250	125	125
EAf	250	15.6	500	500	500
Butf	62.5	62.5	125	125	125
Watf	62.5	62.5	125	62.5	125
Vancomycin	1.9	0.5	1.9	1.9	1.9

Results are mean from three sets of experiments, each set in triplicate.

### Antibacterial activity

In this study, the susceptibility of five Gram-positive bacteria and five Gram-negative bacteria towards the extracts was tested using the disc diffusion method. No activity was found against all Gram-negative bacteria tested. Table 3 reports the inhibition zone diameter of the crude extract and its fractions against five Gram-positive bacteria. In general, a larger zone is indicative of antibacterial potency. Streptomycin, vancomycin and oxacillin were used as reference antibiotics. Antibacterial activities were found in crude, EAf, Butf and Watf fractions. The bacteria most susceptible was *M. luteus *(12.8 - 14.3 mm).

The minimum inhibitory concentration (MIC) and minimum bactericidal concentration (MBC) values are summarized in Table 4. The antibacterial activity against the Gram-positive bacteria except *M. luteus*, in decreasing order was Butf ≈ Watf (MIC 62.5-125 μg/ml; MBC 62.5-125 μg/ml) > EAf (MIC 250 μg/ml; MBC 250-500 μg/ml). Against *M. Luteus *it is of interest to note that EAf and Butf have lowest MIC and MBC, indicating relatively strong activity, although these extracts are generally weaker against the other bacteria (MIC and MBC 250-500 μg/ml). Both Butf and Watf have similar minimum inhibition concentration against *B. cereus*, MSSA and MRSA (MIC 62.5 μg/ml).

### Phytochemical screening

Phytochemical tests for the ethyl acetate, butanol and water fractions showed absence of alkaloids and saponins. All fractions tested positive for flavonoids, terpenoids and tannins (Table 5).

**Table 5:** Phytochemical analysis of *Blechnum orientale *active fractions

Plant constituent	EAf	Butf	Watf
Alkaloids	-	-	-
Saponins	-	-	-
Flavonoids	++	++	++
Terpenoids	+	+	+
Tannins	+	++	+++

+++ Strong; ++ medium; + poor presence; - absent; classification was based on observation of colour intensity and amount of precipitate

## Discussion

In recent years, the search for phytochemicals possessing antioxidant, anticancer and antimicrobial activities have been on the rise due to their potential use in the therapy of various chronic and infectious diseases. Epidemiological and experimental studies have implicated oxidative cellular damage arising from an imbalance between free radical generating and scavenging systems as the primary cause of cardiovascular disease, cancer and aging [2]. Due to risk of adverse effects encountered with the use of synthetic antibiotics, medicinal plants may offer an alternative source for antimicrobial agent with significant activity against pathogenic and infective microorganisms. In addition, a number of antibiotics have lost their effectiveness due to the development of resistant strains, mostly through the expression of resistance genes [18].

Results of our studies confirmed the use of *B. orientale *as a traditional medicine. We found strong antioxidant, cytotoxic and antibacterial activities specifically in the moderately polar fractions EAf and Butf, as well as the polar fraction Watf. High TPC values found in EAf and Butf (675 - 804 mg GAE/g) imply the role of phenolic compounds in contributing these activities. Plant phenolic compounds have been found to possess potent antioxidant [6,13,19-22], antimicrobial [4,23,24] and anticancer activities [5,25]. Phytochemical analysis revealed presence of flavonoids in the active fractions. This finding corroborates with an early study [26] that reported derivatives of flavones (luteolin, apigenin, acacetin and genkwanin) and flavonols (quercetin, kaempferol, isorhamnetin) in this fern. These flavonoids have been found to possess antioxidant, antitumor and/or antimicrobial properties in various studies [24,25,27-31]. Strong presence of tannins in Watf may explain its potent bioactivities as tannins are known to possess potent antioxidant [32], antimicrobial activity [4,33] and anticancer properties [34]. Tannins exert the antimicrobial action by precipitating the microbial protein [33].

Our results on the antioxidant activity showed potential use of this fern as its activities were comparable with the standard antioxidants Trolox-C and α-tocopherol, while exceeding those for some medicinal plants: *Phaulopsis fascisepala *has lower scavenging activity compared to α-tocopherol and BHT [19]; the medicinal King fern *Angiopteris evecta*, IC _50 _> 90 μg/ml [20]; nine selected medicinal plants used in the Indian Traditional System, IC _50_83-560 μg/ml [21] and six medicinal ferns used in Chinese Traditional System, IC _50_27-400 μg/ml [22].

In the evaluation on the anticancer property of this fern, selective cytotoxicity was exhibited against human colon cancer cells HT-29 and HCT-116, while no effect was detected against MCF-7, K562 and liver Chang cells. Due to the selectivity towards colon cancer cells, we chose curcumin as a positive control as it is currently widely used in clinical trials for chemoprevention of colon cancer [35,36]. Amongst the fractions, the butanol fraction displayed the most potential (IC_50 _27.5 μg/ml) although weaker than curcumin (IC_50 _5.4 μg/ml). The impure nature of the Butf may have reduced the effect of the potential anticancer component present in that fraction which if isolated, may then improve its IC_50_. Nevertheless, the IC _50_value of Butf satisfies the criteria established by the American National Cancer Institute (NCI) of cytotoxicity activity, which is an IC_50 _*<*30 μg/ml in the preliminary assay for crude extracts [37]. Our result on the non-cytotoxicity towards MCF-7 (IC _50 _> 100 μg/ml) corroborates that reported previously on this fern [38]. It is known that the use of chemotherapeutic drugs in cancer may cause detrimental effects to normal cells, hence there is a need in the search for drugs which selectively act on tumor cells. Non-malignant Chang cells have been used in cytotoxicity studies to test the effects of drugs/agents on normal cells. For example, two chemotherapeutic compounds against HepG2 cells, eurycomanone and tamoxifen, are reported to be cytotoxic towards Chang cells [39]. Curcumin has also been reported to induce cell death in normal cells such as rat thymocytes and human T cells [40]. Therefore, the selective cytotoxicity shown by the fractions towards HT-29 and non-cytotoxicity toward the liver Chang cells indicates a promising potential for Butf to be developed for use in colon cancer therapy.

Our results on the antibacterial activity confirmed the traditional use of *B. orientale *in the treatment of skin diseases, urinary bladder and stomach discomforts. Its bactericidal activity against *B. cereus*, *M. luteus*, MSSA, MRSA and *S. epidermidis *showed potential use of this fern for the treatment of infectious diseases caused by these bacteria, which have been reported to be skin and food-borne pathogens in various diseases [41-44]. MRSA, probably the most challenging pathogen affecting patients worldwide, is resistant to β-lactams antibiotics used in the treatment of staphylococcal infections [45]. When compared with earlier reports on the antibacterial activity of this fern, we found a few similarities as well as discrepancies. While we found strong activity against *M luteus*, Nick *et al*. [9] reported no activity. The inactivity against *K pneumonia*, *E. coli, P. aeruginosa *was also reported by Banerjee and Sen [46] but in contrast with that reported by Maridass and Ghantikumar [8]. The bactericidal activity of this fern against *S aureus *has also been reported by Maridass and Ghantikumar [8]. The discrepancies in the results could be due to a few factors such as different extracts and methods used, parts of the plant examined, variation in ecological factors, genetic changes, state of maturity and the strains of test microorganisms [47].

The inactivity against all Gram-negative bacteria tested could be due to the impermeable nature of the outer membrane of the bacteria [48]. When compared with some medicinal plants such as *Curcuma longa *L. (MIC 1000-6400 μg/ml) [49] and *Quercus infectoria*(MIC 130 - 1000 μg/ml) [50], our result on anti-MRSA potency of this fern (MIC 62.5 μg/ml; MBC 62.5-125 μg/ml in Butf and Watf) is very promising. Nevertheless, its activity is weak compared to the reference, vancomycin (MIC 1.9 μg/ml). It should be noted that the impure nature of the active fractions may have diluted its efficacy as compared to a pure antibiotic compound. Stronger antibacterial activity against *B. cereus *and *S. aureus *(MIC 62.5 μg/mL) were found in the Butf and Watf fractions compared to those reported in *Grewia occidentalis*, *Polystichum pungens*, *Cheilanthes viridis* and *Malva parvifolia *(MIC 500-5000 μg/ml) [51], *Anethum graveolens*, *Foeniculum vulgare* and *Trachyspermum ammi *(MIC 20-80 mg/ml) [4] while its activity was comparable to some selected Yemeni medicinal plants MIC < 250 μg/ml [5]. Our result concerning the strongest antibacterial activity in the water fraction corroborates those of Dahot [52] who showed that the aqueous extracts of the plant leaves investigated showed significant antimicrobial activity, compared to the other organic counterparts.

## Conclusions

Strong antioxidant, antibacterial and anticancer properties were confirmed in the ethyl acetate, butanol and water fractions. These activities may be due to the strong occurrence of polyphenolic compounds such as flavonoids and tannins. The antioxidant scavenging activity was comparable to those of Trolox-C and α-tocopherol. Selective cytotoxic activity was detected against colon cancer cells HT-29 while noncytotoxic effect to liver Chang cells was established. The water and butanol fractions showed significantly strong bactericidal activity against all Gram-positive bacteria tested, with special reference to MRSA. These findings provide scientific evidence to support its traditional medicinal uses and indicate a promising potential for the development of an antioxidant, anticancer and antibacterial agent from this plant.

## Competing interests

The authors declare that they have no competing interests.

## Authors' contributions

HYL carried out the experimentation as part of PhD study and drafted the manuscript. YYL supervised the work, evaluated the data and corrected the manuscript for publication. KHK supervised the work and evaluated the data on the cytotoxicity study. All authors read and approved the final manuscript.

## Pre-publication history

The pre-publication history for this paper can be accessed here:

http://www.biomedcentral.com/1472-6882/10/15/prepub
